# Mechanisms underlying the autonomic modulation of ventricular fibrillation initiation—tentative prophylactic properties of vagus nerve stimulation on malignant arrhythmias in heart failure

**DOI:** 10.1007/s10741-012-9314-2

**Published:** 2012-06-08

**Authors:** Kieran E. Brack, James Winter, G. André Ng

**Affiliations:** 1 Cardiology Group, Department of Cardiovascular Sciences, University of Leicester, Leicester, LE3 9QP UK; 2 Leicester NIHR Biomedical Research Unit in Cardiovascular Disease, Glenfield Hospital, Clinical Sciences Wing, Leicester, LE3 9QP UK

**Keywords:** Vagus nerve stimulation, Heart failure, Ventricular fibrillation, Nitric oxide

## Abstract

Classical physiology teaches that vagal post-ganglionic nerves modulate the heart via acetylcholine acting at muscarinic receptors, whilst it is accepted that vagus nerve stimulation (VNS) slows heart rate, atrioventricular conduction and decreases atrial contraction; there is continued controversy as to whether the vagus has any significant direct effect on ventricular performance. Despite this, there is a significant body of evidence from experimental and clinical studies, demonstrating that the vagus nerve has an anti-arrhythmic action, protecting against induced and spontaneously occurring ventricular arrhythmias. Over 100 years ago Einbrodt first demonstrated that direct cervical VNS significantly increased the threshold for experimentally induced ventricular fibrillation. A large body of evidence has subsequently been collected supporting the existence of an anti-arrhythmic effect of the vagus on the ventricle. The development of prognostic indicators of heart rate variability and baroreceptor reflex sensitivity—measures of parasympathetic tone and reflex activation respectively—and the more recent interest in chronic VNS therapy are a direct consequence of the earlier experimental studies. Despite this, mechanisms underlying the anti-arrhythmic actions of the vagus nerve have not been fully characterised and are not well understood. This review summarises historical and recently published data to highlight the importance of this powerful endogenous protective phenomenon.

## Sudden cardiac death and the autonomic nervous system

Sudden cardiac death (SCD) resulting from malignant ventricular arrhythmias including ventricular tachycardia (VT) and ventricular fibrillation (VF) represents a significant unsolved clinical problem with an annual death toll of over 325 000 people in the USA (Heart Rhythm Foundation [1]) and around 70,000 in the UK (NICE [2]). The search for prophylactic treatments for SCD remains fundamental and is particularly important in heart failure (HF) which is associated with a significantly increased arrhythmic mortality. A distinctive hallmark of HF is that of autonomic disturbance, that is, increased sympathetic activity and decreased parasympathetic tone. Abnormal autonomic function is been described in all aspects of regulation including alterations to afferent nerve activity, central processing, ganglionic and efferent innervation and alterations to the heart itself. Alterations in autonomic activity arise early in the pathogenesis of HF and appear to precede other stereotypical changes. In a canine tachycardia-induced HF model, vagal tone decreased 3 days after the development of cardiac dysfunction, often preceding the enhancement of sympathetic activity [3, 4]. Activity of the arterial chemoreceptors [5] and cardiac sympathetic afferent fibres [6] are increased, in part, to drive the increase in sympathetic outflow. Enhanced sympathetic activation occurs before the development of symptoms in patients with left ventricular (LV) dysfunction, and reduced parasympathetic drive is present early in patients with symptomatic HF resulting from even mild LV impairment [7].

There is strong evidence that the relationship between impaired neurocardiological control and increased mortality is the result of an increased vulnerability to lethal ventricular arrhythmias [8]. Whilst the clinical and prognostic significance of sympathetic over-activation is well established [9] and forms the rationale for β-blockers as standard HF therapy, parasympathetic regulation and its modulation has received much less attention. There is, however, important evidence illustrating that the vagus nerve has an antiarrhythmic action in the ventricle. The mechanisms by which the autonomic nervous system, especially its parasympathetic branch, influences ventricular arrhythmias are poorly understood and SCD remains a significant clinical problem. Advances in our ability to treat HF have allowed for significant improvements in patient survival; however, a significant proportion of patients still die suddenly as a result of malignant arrhythmias. Whilst milder forms of heart failure (NYHAs II and III) are associated with lower mortality compared to NYHA IV, the proportion of overall mortality attributable to lethal ventricular arrhythmias is higher [10]. Although there is no direct data exploring this notion, it is tempting to postulate that measures which improve HF status, whilst reducing overall mortality (with pump failure death as main contributor), paradoxically convert patients into a ‘more arrhythmogenic’ HF phenotype. To achieve further improvements in patient survival, new therapeutic strategies are required to address this. The vagus nerve could provide a powerful endogenous anti-arrhythmic means to achieve such an end. This review aims to discuss historical and recent developments in our understanding of the anti-arrhythmic influence of the vagus on the ventricle focussing on the mechanisms of its protective effect. The use of vagus nerve stimulation (VNS) for the prevention of malignant ventricular arrhythmias in HF will be discussed—relating phenotypical changes associated with the syndrome with data on its mechanisms of action.

## Parasympathetic tone and patient prognosis

There is a strong correlation between survival and ‘markers’ of parasympathetic tone in patients with HF irrespective of β-blocker therapy [11–13] or following myocardial infarction (MI) [14]. Due to the invasive nature of the direct measurement of vagal activity, studies rely on indirect measurements of sinus node behaviour in the form of heart rate variability (HRV) and baroreceptor reflex sensitivity (BRS). HRV is a measure of the variability of time interval between heart beats (RR interval) and reflects the activity of sympathetic and parasympathetic branches. Several measures of HRV including the standard deviation of the RR interval (SDNN) [15] and the low-frequency component on power spectral analysis [16] are independent risk markers. BRS measures changes in heart rate in response to pressor doses of phenylephrine and/or vasodilatory sodium nitroprusside (SNP) reflecting a reflex pathway primarily involving the parasympathetic nervous system [17, 18]. BRS is calculated from plots of heart rate or RR interval as a function of the change in blood pressure, the slope of the relationship provides the BRS. The predictive power of indirect measures of parasympathetic activity was first demonstrated in animal studies. A reduction in BRS correlates with an increased occurrence of SCD in animals with healed MI subjected to exercise and acute coronary artery occlusion (CAO) [18, 19]. Larger studies subsequently revealed BRS to be a strong predictive marker both before MI and after MI, where a depressed BRS predicts a higher risk [20]. Studies of single cardiac vagal fibre activity reveal an increase in vagal activity immediately following CAO occurred in animals destined to survive and that depressed vagally activity in response to changes in blood pressure predicted an increased risk of SCD post-MI [21].

Several small- and large-scale clinical trials have demonstrated the predictive value of BRS and HRV in patient populations. An early prospective clinical study reported that a low BRS was significantly associated with greater all-cause mortality [17], and a subsequent follow-up trial found a strong association between BRS and the occurrence of malignant arrhythmic events but not with all-cause mortality [22]. Several time domain measures of HRV are also reported to be powerful predictor of all-cause mortality following MI [15, 23–25]. Low-frequency (typically < 0.04 Hz) power from spectral analysis of RR intervals reflects vagal tone, and its reduction correlates with all-cause mortality and arrhythmic death independent of other factors, for example, left ventricular ejection fraction [16]. The prognostic value of BRS and HRV has been confirmed in large-scale (500–1,000 + patients) randomised clinical trials conducted in patients post-MI (ATRAMI [14]) and in patients with chronic HF (UK-HEART [13]).

## The parasympathetic nervous system and ventricular arrhythmias

**Fig. 1:**
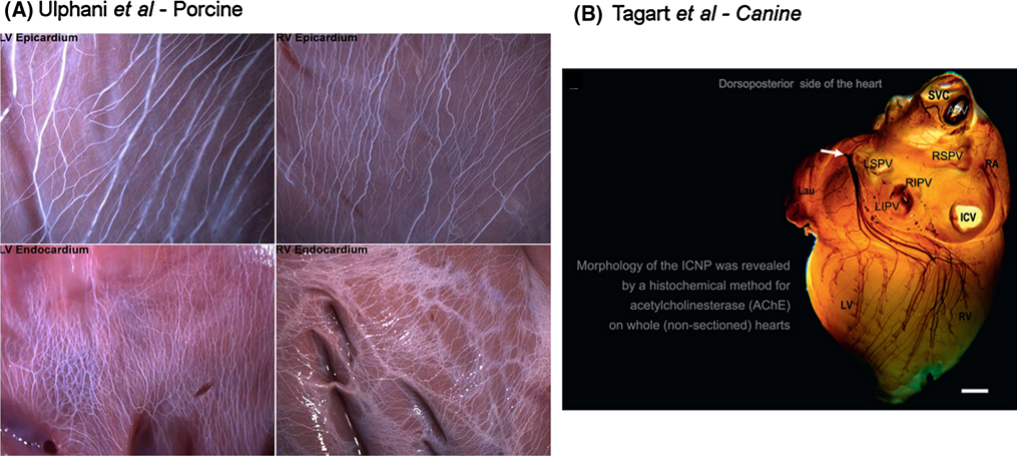
Parasympathetic innervation of the mammalian ventricles. A composite of images demonstrating extensive parasympathetic innervation of the ventricles of the pig and dog. **a** Acetylthicholine precipitation demonstrating the gross morphology of parasympathetic fibres innervating the epi- and endocardial regions of the porcine ventricles. **b** Histochemical staining of acetylcholinesterase containing nerve fibres in the 1-month-old canine heart. Reproduced with permission from Ulphani et al. [33] and Taggart et al. [150] respectively

The dogma of physiology teaching states that vagal post-ganglionic nerves modulate the heart through acetylcholine (ACh) acting on muscarinic receptors (mAChRs) to slow heart rate, increase atrioventricular conduction and inhibit atrial force, with little influence on ventricular performance. The latter reflects historical reports that ventricular parasympathetic (cholinergic) innervation was sparse. This view is, however, outdated because modern histological techniques reveal a dense and intricate network of ACh containing nerves running over the epi- and endo-cardial surfaces of left and right ventricles and a widespread distribution of mAChRs throughout the ventricle [26]. More importantly, it is worth reminding readers that the vagal-cholinergic pathway is capable of inhibiting the strong inotropic response to adrenergic activation [27] and as such has a clear functional role. Dense parasympathetic innervation is demonstrated in all species studied including the mouse [28, 29], guinea pig [30], cat [31], dog [32], pig [33], sheep [34] and human ventricle [35, 36] (see Fig. 1).

## Parasympathetic innervation and receptors

The somata of the parasympathetic efferent pre-ganglionic neurones arise chiefly from the ventral lateral region of the nucleus ambiguous, and a smaller proportion from the dorsal motor nucleus and intermediate zone between these sites [37]. Pre-ganglionic fibres are carried within the Xth cranial nerve, that is, the vagi and converge at several atrial and ventricular ganglionated plexuses which regulate cardiac function through post-ganglionic projections post-ganglionic in the atrial and ventricular tissues. Significant laterality is noted in the effects of the two vagi with the right vagus, exerting a greater influence on the sinoatrial node than the left vagus [39, 40] with a greater influence of the left vagus on atrioventricular conduction in the rabbit [38], although significant overlap exists [41]. The existence of laterality is clinically important for the application of VNS where excessive bradycardia should be avoided.

There is a complex network of neuronal subtypes within the ganglionated plexuses which can modulate the heart on a beat-by-beat basis that can act independent of peripheral input [40]. The role of this *heart brain*, reviewed by Armour [41], is an emerging and fascinating area of research with potential implications in the autonomic modulation of both atrial and ventricular arrhythmias. This is highlighted by evidence that neurochemical stimulation of decentralised intrinsic cardiac neurones can generate atrial and ventricular arrhythmias [42] and that reduced vagal tone in HF may be directly attributable to changes in ganglionic function [43].

Post-ganglionic vagal fibres are primarily cholinergic in nature releasing ACh which act on mAChRs. Five distinct mAChRs subtypes (M_1_–M_5_) have been described, and 3 subtypes are found within the heart (M_2_, M_3_, and M_4_) [44–46]. The most abundant mAChR is the Gi-protein-coupled M_2_ receptor and is found on cardiomyocytes [44] and within intracardiac ganglia [47]. Evidence from receptor knockout mice suggests that the M_2_ receptor subtype is predominantly responsible for regulating cardiac function [45] through a G_i_-protein mediated direct action on ACh-dependent ion channels such as IK_Ach_ and indirect effects on inhibition of adenylate cyclase activity that reduces intracellular cAMP. cAMP is known to stimulate the non-selective cationic current (*I*_*f*_) which drives spontaneous depolarisation of pacemaker tissues such as the sinoatrial and atrioventricular nodes and purkinje fibres. The activation of the IK_ACh_ results in membrane hyperpolarisation, increasing the cells threshold for firing which contribute to heart rate reduction. M_3_ and M_4_ ACh receptors are [46] preferentially linked with Gq-proteins. The role of the M3 receptor in regulating cardiac function, especially electrophysiology, is discussed later. In addition to ACh, several peptides are co-localised within cholinergic neurones. The most common is vasoactive intestinal peptide (VIP) which is considered to be positively inotropic by increasing adenylate cyclise activity, although this is contentious [48]. It is therefore possible that VIP interacts with ACh at the post-junctional level, opposing the inhibitory actions of ACh on intracellular cAMP accumulation.

## Vagal modulation of arrhythmia susceptibility

Table 1 presents a summary of studies investigating the effects of VNS on the occurrence of ventricular arrhythmias under a number of conditions. This table aims to summarise the methodological approaches used in each study and to present the results of each study in a condensed but comprehensive format.

**Table 1:** Overview of studies looking at the effects of vagal nerve stimulation on ventricular arrhythmias in animal models

Authors and species	Experimental conditions		Protocols				Factors investigated									Results
	Mode	ANS	VFT	CAO	I/R	Sp	VNS	Rt	AA	HR	MB	ΒΒ	GJ	Vag	NO	
*Normal animals*																
Sherlag et al. (1970) [50] Dog	In vivo	✓ Sym ✓ Para ✓ Circ		✓		✓	✓									VNS interrupted the occurrence of polymorphic VT following CAO
Goldstein et al. [51] Dog	In vivo	✓ Sym ✓ Para ✓ Circ		✓		✓	✓			✓	✓					MB ↑ arrhythmia occurrence ↓ Arrhythmias in animals with reflex ↓ HR during CAO ↔ Control vs. VNS arrhythmic events
Myers et al. [52] Dog	In vivo	✓ Sym ✓/✗ Para ✓ Circ		✓		✓	✓			✓						Intensity dependent ↓ in arrhythmic events Responses ↔ during constant pacing Responses ↔ following decentralisation
Kolman et al. [54] Dog	In vivo	✗ Sym ✗ Para ✓ Circ	✓				✓					✓				↔ VFT with VNS alone (decentralised vagi) ↓ VFT during SNS blocked by BB ↑ VFT to control during SNS-VNS combined
Yoon et al. [55] Dog	In vivo	✓ Sym ✓/✗ Para ✓ Circ	✓	✓			✓				✓	✓				↑ VFT with VNS in normal ↔ during CAO (decentralised vagi) ↓ VFT with MB in normal ↔ during CAO ↑ VFT during BB ↔ VFT with VNS or MB following BB
Kent et al. [56] Dog	In vivo	✓ Sym ? Para ✓ Circ	✓	✓			✓			✓		✓				VFT an inverse function of HR during CAO but not in normal animals HR independent ↑ VFT during VNS
Corr and Gillis [57] Cat	In vivo	✓ Sym ✓/✗ Para ✓ Circ		✓		✓	✓				✓			✓		↑ In HR and VF death during CAO following bilateral vagotomy and MB ↔ VF death during HR matched pacing (HR independent effect)
Zuanetti et al. [57] Cat	In vivo	✓ Sym ✓/✗ Para ✗ Circ			✓	✓	✓		✓	✓				✓		↔ In arrhythmic events following vagotomy ↓ Arrhythmic events during VNS ↑ Arrhythmic events during baseline matched pacing vs. VNS HR independent ↓ arrhythmia vs. control
Ng et al. [68] Rabbit	In vitro	✗ Sym ✗ Para ✗ Circ	✓				✓	✓		✓	✓					↓ Restitution slope, ↑ ERP and ↑ VFT during VNS ↓ Alternans range during rapid pacing with VNS HR independent ↓ Restitution slope, ↑ ERP and ↑ VFT during VNS
Brack et al. [131] Rabbit	In vitro	✗ Sym ✗ Para ✗ Circ	✓				✓	✓							✓	↓ Slope and ↑ VFT abolished by NOS inhibition Response restored by l-Arginine supplementation ↑ ERP during VNS unaffected by NOS inhibition
Brack et al. [104] Rabbit	In vitro	✗ Sym ✗ Para ✗ Circ	✓				✓	✓			✓				✓	↑ NO-dependent fluorescence during VNS ↓ Restitution slope, ↑ ERP and ↑ VFT during VNS ↔ Response to VNS during MB ↔ Response to VNS during endothelial denudation
Ando et al. [111] Rat	In vivo	✓ Sym ✓ Para ✓ Circ		✓		✓	✓				✓		✓			↓ Arrhythmic events during CAO with VNS VNS effects abolished by atropine Pre-conditioning protective effect of VNS ↓ Cx43 expression at intercalated discs following CAO Normalisation of connexion-43 expression by VNS
Wang et al. [110] Rat	In vivo	✓ Sym ✓ Para ✓ Circ		✓		✓					✓ M3					↓ CAO arrhythmic events with muscarinic agonist Protection abolished following M3 MB M3 activation abolished Ca^2+^ overload in isolated ventricular myocytes
*Chronic MI and HF*																
Schwartz et al. [59] Dog	In vivo *4* *weeks post-MI*	✓ Sym ✓ Para ✓ Circ		✓		✓										50 % of post-MI animals develop arrhythmias during following exercise testing and CAO Evidence for reduced arrhythmic risk in animals with a ↓ in HR during COA
Schwartz et al. [20] Dog	In vivo *4* *weeks post-MI*	✓ Sym ✓ Para ✓ Circ		✓		✓										BRS lower in animals susceptible to exercise and CAO-induced arrhythmia Low BRS associated with a ↑ Risk of SCD
De Ferrari et al. [19] Dog	In vivo *4* *weeks post-MI*	✓ Sym ✓ Para ✓ Circ		✓		✓					✓					↑ Arrhythmia occurrence during exercise and CAO testing during MB Weak symp and strong para reflexes associated with improved survival and ↓ arrhythmic events
Vanoli et al. [60] Dog	In vivo *4* *weeks post-MI*	✓ Sym ✓ Para ✓ Circ		✓		✓										↓ Arrhythmia occurrence during exercise and CAO with VNS (returned to baseline following cessation) Heart rate-dependent and independent protection
Zheng et al. [75] Rat	In vivo 4-5 months post-MI	✓ Sym ✓ Para ✓ Circ				✓	✓									↑ PVCs post-MI Complete abolishment of PVCs by VNS ↑ PVCs following cessation of VNS
Sabbah et al. [83] Dog	In vivo	✓ Sym ✓ Para ✓ Circ											✓			↓ Cx43 expression in failing heart Normalised Cx43 expression following chronic VNS

*AA* accentuated antagonism, *ANS* state of autonomic nervous system, *BB* β-adrenoceptor blockade, *BRS* baroreceptor reflex sensitivity, *CAO* coronary artery occlusion, *Circ* circulating neurohumoral factors, *Cx43* connexin-43, *GJ* gap junction, *HR* heart rate dependency, *M3* muscarinic receptor 3, *MB* muscarinic receptor blockade, *NO* nitric oxide, *Para* background parasympathetic nervous system drive, *Rt* restitution, *Sym* background sympathetic nervous system drive, *VFT* ventricular fibrillation threshold, *VNS* vagus nerve stimulation

? = Unknown/unclear methodology, ✓(protocols and factors) = studied, ✓(experimental conditions) = intact/present, ✗ (experimental conditions) = not present, ✓/✗(experimental conditions) = adjusted as per experimental methodology, ↑ = increased, ↓ = decreased, ↔ = unchanged

### Historical perspective

The first report that VNS reduced the susceptibility of the ventricle to experimental arrhythmia was presented by Einbrodt in 1859 [49], who demonstrated that VF was harder to induce in the dog during stimulation of the vagus. The anti-arrhythmic action of VNS was later reproduced by Scherlag et al. [50] who showed that VNS interrupted the spontaneous occurrence of VT during CAO. Studies by Goldstein et al. [51], Myers et al. [52] and several others [53–56] in the 1970s confirmed the anti-arrhythmic action of VNS. Several studies provide evidence that VNS is protective against experimental-induced VF using undiseased hearts [54–56]. Kent et al. [56] demonstrated a VNS-dependent increase in the current required to induce VF, using a train of electrical pulses applied during the refractory period, that is, the ventricular fibrillation threshold (VFT), in the anaesthetised dog. Yoon et al. [55] similarly demonstrated a significant increase in VFT during VNS which was not maintained in the presence of the non-selective β-adrenergic antagonist propranolol. Similarly, a study by Kolman et al. [54], in which the left stellate ganglion was crushed, demonstrated no anti-arrhythmic effect in control conditions but found significant protection when VNS was applied after stimulation of sympathetic nerves suggesting significant sympathetic–parasympathetic interactions in the anti-arrhythmic action of VNS, which are discussed in later.

A number of studies have shown that VNS prevents arrhythmias as a result of CAO. Myers et al. [52] investigated the effects of low- and high-frequency VNS on spontaneous VF during acute CAO. Vagal stimulation was associated with an intensity-dependent reduction in VF occurrence that was coupled with higher survival rates. These data are supported by Kent et al. [56]. In the converse experiment, Corr and Gillis [57] found that bilateral vagotomy and atropine perfusion increased CAO-induced mortality, suggesting that intrinsic vagal activity was protective. In contrast, Yoon et al. [55] reported no effect of vagal stimulation on VFT in the ischaemic dog heart despite a pronounced response in non-ischaemic controls. A study conducted by Zuanetti et al. [58] demonstrated effects of bilateral vagotomy and of VNS on the occurrence of malignant ventricular arrhythmias (VF or fast sustained VT) following reperfusion of the ischaemic feline myocardium—a reduction in the incidence of both VF and fast sustained VT occurred during VNS whilst bilateral vagotomy resulted in a 55 % reduction in arrhythmia occurrence when compared to unoperated controls.

### Studies in a canine model of SCD

An elegant canine model of SCD developed by Peter Schwartz and colleagues has been used extensively to study the protective effect of the vagus in conscious animals aimed at circumventing the confounding influence of anaesthetics [19, 20, 59, 60]. Animals are subjected to ligation of the left anterior descending coronary artery, instrumented for future studies and subsequently allowed to recover for 1 month. Surviving animals go onto a treadmill exercise test with progressively increasing workload together with CAO of the left circumflex artery from the start of the last minute of the exercise regime for a period of 2 min resulting in the reproducible occurrence of VF in 50–60 % of animals [20, 59]. An ischaemia-induced reduction in heart rate in those dogs which survived suggests that active vagal reflexes protect from malignant ventricular arrhythmias. De Ferrari et al. [19] later studied vagal tone in these animals. The authors found that muscarinic blockade with atropine promoted premature ventricular contractions, VT and VF in animals with no arrhythmia during control. The activation of strong vagal reflexes (i.e. HR reduction following CAO) was shown to be important for survival in 25 % of animals studied and was significantly correlated with measures of BRS. Vanoli et al. [60] studied the effects of direct VNS on the occurrence of SCD in dogs that survived MI. Right VNS, applied before and throughout CAO, reduced the occurrence of VF from 92 % in control to 10 % during VNS. These data provide strong supportive evidence of the important anti-arrhythmic protection of the vagus nerve on the ventricle.

### Studies in the isolated innervated rabbit heart

**Fig. 2:**
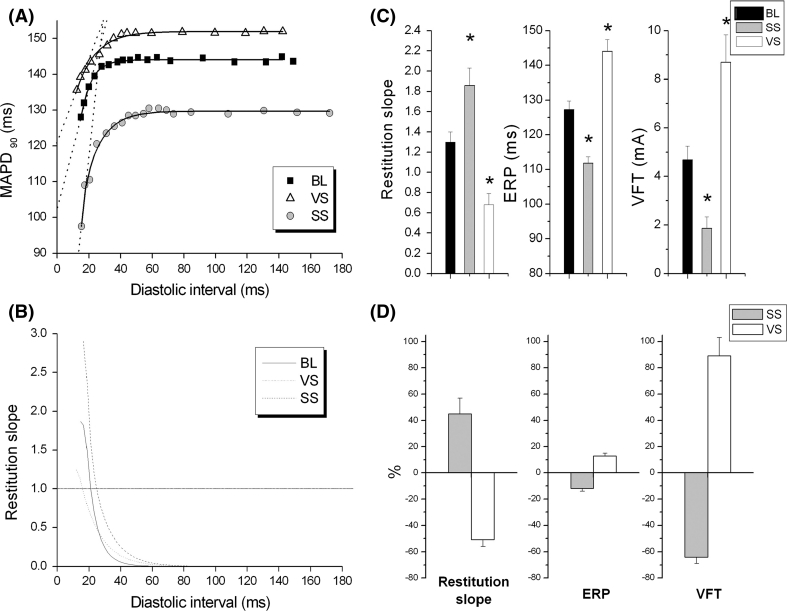
Autonomic modulation of electrical restitution and ventricular fibrillation threshold. The effects of bilateral sympathetic (SS) and parasympathetic (VS) nerve stimulation on the slope of the electrical restitution curve, effective refractory period (ERP) and ventricular fibrillation threshold (VFT) of the isolated innervated rabbit heart. **a** and **b** Data from a single experiment demonstrating the effect of SS and VS on the electrical restitution curve. **c** and **d** Mean data on the effects of SS and VS on the restitution slope, ERP and VFT. *BL* baseline. *P < 0.05; comparison to baseline. Reproduced with permission from Ng et al. [68]

The onset of VF is believed to be associated with the break-up of spiral waves or rotors into multiple wavelets and oscillations in electrical activity [61]. One mechanism considered key in the development of these oscillations is electrical restitution. Electrical restitution is the relationship between action potential duration (APD) preceding diastolic interval (DI). APD is relatively long at slow heart rates (long DI) but shortens with high heart rates or an ectopic beat (short DI). The magnitude of APD shortening with changes in DI characterises the dynamics of restitution which can be described by the maximum slope during restitution curve (plot of APD vs. DI). The ‘restitution hypothesis’ proposes that the slope of the APD restitution curve is related to the susceptibility to arrhythmia initiation. Typically oscillations are facilitated when the slope of the APD restitution curve is >1 [62]. In situations with a steep restitution curve, a small change in DI leads to a large change in APD and hence dynamic instability and is supported from data from mathematical [63, 64] and biological studies [62, 65] relating to VF vulnerability [66, 67]. In addition, there is supportive evidence from drugs that reduce restitution slope also prevent VF [65]. Using the isolated innervated rabbit heart preparation, we investigated the effects of VNS on electrical restitution and ventricular fibrillation susceptibility [68]. VNS caused a flattening of the electrical restitution slope, an increase in the current required to induce ventricular fibrillation (increased VFT) alongside a prolongation of MAPD and ERP (see Fig. 2). VFT was inversely correlated with the slope of the electrical restitution curve, in keeping with the restitution hypothesis that predicts an increase in arrhythmia susceptibility as the slope increases. The anti-fibrillatory effect of the vagus nerve occurs in the absence of any background sympathetic activity [38], suggesting a *direct* anti-arrhythmic effect which is separate from the indirect action concerning antagonism of adrenergic effects (accentuated antagonism), which is evident from single cells studies using pharmacology investigating the effects on individual ionic currents from heart failure preparations [69].

## Spontaneous versus induced arrhythmia

The use of ‘spontaneous’ and induced methods of arrhythmia occurrence is a contentious issue that warrants discussion. CAO represents one of the most common methodologies to generate arrhythmias which rely on spontaneous arrhythmia generation. Studies utilising CAO often use quantitative measures of arrhythmic events, that is, number of premature ventricular contractions (PVCs), VT and VF or the time course of these events. A disadvantage of this approach is the stochastic nature of arrhythmic event occurrence. The development of the canine model of SCD addressed many of the issues of reproducibility [59]. However, this method is rather time-consuming, expensive, technically difficult and is highly dependent upon multiple factors, that is, ischaemia and adrenergic activation. Whilst this model could be viewed as physiologically relevant, the assessment and investigation of the *direct* effects of VNS are not possible. VFT provides a quantifiable and robust measure of arrhythmia susceptibility, allowing that the examination of how physiological, pathophysiological and pharmacological factors can affect electrical stability in addition to effects from nerve stimulation. VFT is widely used for the study of arrhythmia susceptibility and has been shown to predict the increased and decreased susceptibility of the ventricle to arrhythmias in a range of situations [56, 66, 70]. Some investigators are concerned that VFT does not predict the anti-arrhythmic actions of certain drugs and fails to predict the occurrence of spontaneous arrhythmias [71]. However, Harumi et al. [72] have demonstrated that anti-arrhythmic drugs can be classified depending upon their effects on VFT, ventricular refractoriness and electrical restitution properties, indicative of the multiple mechanisms of action of many of these drugs. Despite these limitations, VFT appears to be a reliable indicator for the effects of the autonomic nervous system on ventricular arrhythmias [55, 56].

## Vagus nerve-mediated protection from malignant arrhythmias in heart failure

Data on the use of vagal stimulation in the treatment for arrhythmia secondary to HF are somewhat limited despite the fact that ventricular arrhythmias are responsible for a large proportion of SCDs and that arrhythmic disturbance, that is, ectopic beats or runs of non-sustained ventricular tachycardia, occurs in 50–80 % of all HF patients [73, 74]. The effects of chronic vagal stimulation on arrhythmias occurrence in conscious rats with MI-induced HF were investigated by Zheng et al. [75]. Right cervical VNS conducted 3–5 months post-MI for a period of 7 days prevented the characteristic occurrence of premature ventricular and supraventricular contractions. Sabbah [76] reported on the effects of chronic VNS in dogs with microembolism-induced HF. In these studies, VNS was applied using a negative-feedback loop to maintain heart rate at a level 10 % below baseline for a 3-month period. While the main focus of this study was to look at the effect of VNS on haemodynamics and ventricular remodelling, the effect of VNS on gap junction protein expression was documented. HF causes translocation of the gap junction protein connexin away from the intercalated discs which regulate cell-to-cell excitability, allowing the passage of small ions between adjacent cardiomyocytes. Disruptions to this cell-to-cell coupling play an important role in arrhythmogenesis and underpin conduction through the myocardium. Reduced expression of connexin-43, the primary form expressed in the ventricular myocardium, slows ventricular conduction and increases the dispersion of transmural APD which is linked to the increased incidence of tachyarrhythmias and SCD [77, 78]. VNS therapy reverses the reduction in connexin-43 mRNA and protein expression associated with HF [76], providing strong supportive, yet indirect, evidence that VNS may have anti-arrhythmogenic properties in the failing heart. The effects of VNS can be mimicked by the use of pharmacological agents. Pyridostigmine, a cholinesterase inhibitor, decreases the rate of ACh degradation at the post-ganglionic synapse and prevents the occurrence of single ventricular ectopic beats in patients with HF NYHA class I to III and an ejection fraction of <45 %, although no effect was noted on paired ectopic beats or on the occurrence of periods of ventricular tachycardia [79]. The suppression of cardiac arrhythmias in congestive HF by clonidine [80], a centrally acting α2 adrenoceptor agonist which acts to suppress sympathetic nervous activity, demonstrates how VNS could be used to antagonise the pro-arrhythmic influence of the sympathetic nervous system. Despite limited data on the effects of VNS on arrhythmia susceptibility, there is strong evidence that VNS improves autonomic balance in HF [81, 82], reducing sympathetic hyperactivity and increasing vagal tone, highlighting its potential as an anti-arrhythmic therapy.

In addition to its anti-arrhythmic properties, chronic VNS has been shown to reverse the contractile dysfunction and adverse remodelling associated with experimental HF [81–84] and appears to be beneficial even when applied at subthreshold intensities insufficient to cause changes in HR [85]. The clinical application of chronic vagal therapy is recently described by De Ferrari and Schwartz [85] and will not be discussed in detail here. It is worthy to note that the profound and beneficial effects of the vagus nerve on the cardiovascular system have prompted a number of clinical trials (i.e. INOVATE-HF and NECTAR-HF), examining the application of chronic VNS in the treatment for HF [85]. There is clearly a need for further study on the potential benefit of VNS in the prevention of arrhythmias in HF, especially given that arrhythmia-related deaths remain a significant clinical burden in spite of advances in treatment [10]. Robust clinical trials will be required to truly assess the role of VNS in this regard, but it is possible to speculate how VNS may be beneficial in the treatment for HF on the basis of what we know about the ‘disease’ process and its phenotypical characteristics.

## Mechanisms of the anti-arrhythmic effect of the vagus nerve

Vagal stimulation modulates ventricular arrhythmias by a number of direct and indirect actions including activation of muscarinic receptors, antagonism of sympathetic actions, heart rate reduction, prolonged APD and reduced APD dispersion, effects on restitution/refractoriness, and possibly via nitric oxide (NO). Several theories describe how alterations in cardiac electrophysiological parameters may influence the generation and propagation of ventricular arrhythmias. Table 2 summarises some of the tentative mechanisms underlying arrhythmogenesis and how the vagus nerve may act via these factors.

**Table 2:** Tentative mechanisms by which the vagus nerve can exert anti-arrhythmic action on the ventricle

Electrophysiological parameter	Relationship to arrhythmias	Modulation by the vagus nerve
Electrical restitution	↑ Slope of the electrical restitution curve (APD vs. DI) Dynamic Instability at short DI ↑ Arrhythmic risk ↑ Spatial dispersion of restitution ↑ Arrhythmic risk	VNS ↓ electrical restitution slope Direct NO-dependent mechanism ↓ Symp drive ↓ Arrhythmic risk
Refractoriness	↑ Time course of repolarisation in absence of ↑ in APD dispersion and/or conduction velocity ↓ Arrhythmic risk	VNS ↑ ERP Some evidence of uniform prolongation ↓ Arrhythmic risk
APD dispersion	↑ Symp drive Innervation BASE > APEX ↑ APD dispersion ↑ Arrhythmic risk GJ remodelling in ischaemia and HF ↑ Transmural APD dispersion ↑ Arrhythmic risk	VNS ↓ Symp drive ↓ APD Dispersion Direct VNS ↑ APD ↑ ERP and ↓VFT Rate-dependent and independent effects VNS ↓ GJ remodelling Direct M3 MAChR modulation of GJ
Conduction	Gap junction remodelling in ischaemia and HF Slower conduction ↑ Arrhythmic risk	VNS ↓ GJ remodelling Direct M3 MAChR modulation of GJ
Intracellular Ca^2+^ load and DADs	↑ Symp drive ↑ Ca^2+^ load in SR ↑ DADs and spontaneous contractions ↑ Arrhythmic risk	VNS ↓ NA release and cAMP accumulation ↓ SR Ca^2+^ load No evidence of direct effect on Ca^2+^ handling in unstimulated ventricular tissue
Premature ventricular contractions	Substrate and trigger for re-entrant arrhythmias ↓ Pumping efficiency ↑ Symp drive ↑ Arrhythmic risk	VNS ↓ premature ventricular contractions in animals with chronic HF ↓ Arrhythmic risk

*APD* action potential duration, *Ca* ^*2*+^ calcium, *DADs* delayed after depolarisations, *DI* diastolic interval, *ERP* effective refractory period, *HF* heart failure, *MAChR* muscarinic acetylcholine receptor, *NA* noradrenaline, *NO* nitric oxide, *SNS* sympathetic nerve stimulation, *Symp* sympathetic, *VFT* ventricular fibrillation threshold, *VNS* vagus nerve stimulation

### Accentuated antagonism

Interaction between the sympathetic and parasympathetic branches of the autonomic nervous system is complex. Competitive antagonism between autonomic divisions provides an important site of regulation. Samaan [86] first illustrated that vagal stimulation attenuated the chronotropic enhancement associated with sympathetic nerve stimulation (SNS), by demonstrating that the actual change in HR with concomitant VNS and SNS was different to the algebraic sum of the heart rate changes during individual stimulation of each branch. This was later termed ‘accentuated antagonism’ by Levi and Zieske in [87]. These data demonstrate that the vagus has a powerful antagonistic effect on the chronotropic effect of SNS which is confirmed by others [57]. A similar antagonistic action exists in the control of ventricular performance, intracellular calcium handling and cardiac electrophysiology [87–90].

Interaction between the sympathetic and vagal systems occurs via pre- and/or post-junctional mechanisms. ACh binds to mAChRs, on the pre-synaptic sympathetic nerve terminals (M_3_) and M_2_ receptors on cardiomyocytes to inhibit noradrenaline (NA) release and/or accumulation of intracellular cAMP, respectively [89, 91–94]. With respect to arrhythmogenesis, Kolman et al. [54] reported that the beneficial effects of VNS on ventricular arrhythmia vulnerability were entirely attributed to parasympathetic–sympathetic interaction as the protective effect of VNS was abolished by propranolol. Similarly, the anti-arrhythmic effect of VNS in undiseased hearts is abolished by treatment with propranolol [81]. Several investigators have shown a reduction in the anti-arrhythmic effect of VNS following treatment with atropine, supporting the role of muscarinic receptor activation in these studies [77, 81]. It should also be noted that the sympathetic nervous system can regulate the vagal release of ACh and exerts antagonistic effects at the myocyte level through the actions of NA and co-released neuropeptide Y (NPY) [95–103]. How these signalling pathways are involved in arrhythmogenesis remains to be elucidated.

The role of accentuated antagonism in regulating sympathetic outflow to the heart is nothing new; however, it is particularly relevant to the pathogenesis of HF. HF represents a deleterious spiral of events in which cardiac function declines, necessitating an increase in cardiac output which is mediated through and increase in sympathetic activity. The resulting increase in demand, on an already compromised system, results in further deterioration and a greater requirement for increased sympathetic tone to support cardiac output. By our understanding of sympathetic–parasympathetic interaction, if there is an increase in sympathetic activity, there tends to be a reciprocal decrease in parasympathetic tone, as evidenced in studies using HRV and BRS [13, 14]. The beneficial effects of β-blockade therapies in the treatment for HF highlight how antagonising the action of sympathetic nervous system puts a ‘brake’ on this vicious cycle which improves the quality of life and extends life expectantly [10]. β-Blockade not only decreases energetic demand in the heart, but, in theory, could reduce the sympathetic antagonism of the parasympathetic nervous system. There is, therefore, an inherent difficulty in attributing beneficial effects of β-blockade to a single mechanism. In reference to arrhythmias, the benefits appear to be mediated by (1) a reduction in the direct actions of the sympathetic nervous system on the ventricular myocardium, (2) an enhancement of parasympathetic outflow or (3) a combination of these two. As discussed below there are several mechanisms whereby the parasympathetic nervous system directly influences ventricular arrhythmia susceptibility. The use of VNS for the treatment for HF may be viewed as a complementary approach to β-blockade, whereby vagal tone is increased by direct electrical stimulation. The benefit of VNS on protecting against ventricular arrhythmias may occur through (1) a direct action on of the vagus on the ventricle, (2) antagonism of the actions of the SNS or (3) a combination of these factors. The former is supported by our studies in the isolated innervated rabbit heart, demonstrating a significant anti-arrhythmic action of VNS [68] in the absence of underlying sympathetic nervous activity [38].

### Direct muscarinic receptor dependent effects—the M_3_ receptor

Several studies have demonstrated that the beneficial effect of the vagus nerve on the occurrence of VF is related to muscarinic receptor activation. Perfusion of atropine, a non-selective muscarinic receptor antagonist, increased the occurrence of CAO-induced VF in several studies [56], including studies conducted in the canine model of SCD [19]. In contrast, we have found that atropine did not abolish the effect of VNS on the VF inducibility in the isolated innervated rabbit heart [103]. This may reflect differences in the methodology of VF induction or species differences in muscarinic receptor distribution, but more importantly the existence of different cellular mechanisms.

Since the discovery of a functional M_3_ mAChR in the mammalian myocardium by Jaiswal et al. [105], there have been significant advances in our understanding of the roles of M_3_ in the control of cardiovascular physiology. The Gq-protein linked M_3_ mAChR is known to modulate heart rate and cardiac repolarisation, regulate cell-to-cell communication, be beneficial against ischaemic and oxidative injury, and promote or suppress the formation of arrhythmias in a manner dependent on the location of and/or phenotype of arrhythmia, that is, atrial (increased) versus ventricular (decreased) [106]. Pharmacological activation of M_3_ receptors reduces the occurrence of ischaemia-, aconitine- and ouabain-induced ventricular arrhythmias [107]. Further to this, over-expression of the M_3_ mAChR gene reduces the occurrence of spontaneous arrhythmias in a mouse model of ischaemia reperfusion [108]. The role of the M_3_ receptor in regulating cardiac repolarisation and cell-to-cell communication is particularly relevant to the discussion of ventricular arrhythmia susceptibility. The M_3_ receptor activation can stimulate the potassium channel I_KM3_ [109], which has been shown to directly modulate cardiac membrane repolarisation [110]. Studies in isolated cells suggest that M_3_ activation reduces calcium overload in ventricular myocytes, and it has been proposed that this action mediates its protective action in ischaemia-induced arrhythmias [110]. In addition, the M_3_ receptor appears to regulate the expression of phosphorylated connexin-43 (Cx43), a gap junction channel protein highly expressed in ventricular tissue and reduced in ischaemia and HF. Efferent vagal nerve stimulation may protect the heart form ischaemia-induced arrhythmias by preventing a reduction of phosphorylated Cx43 associated with ischaemia [111]. Similarly chronic therapy with VNS appears to normalise the expression of Cx43 within the left ventricular myocardium of dogs with HF [83].

Evidence from studies in gene knockout mice suggests that the effects of M_3_ activation oppose those of the M_2_ receptor, to enhance contractility and increase heart rate [45]. It is recognised that the M_3_ receptor promotes arrhythmias in atrial tissues but suppresses ventricular arrhythmias (a likely result of opposing effects on repolarisation time course), which may indicate difference in the distribution of the mAChRs throughout the myocardium or regional variations in ion channel expression (e.g. greater IK_ACh_ in atrial tissues). Importantly, the expression of M_3_ appears to be increased in hearts from patients with congestive heart failure [106]. Whilst pharmacological evidence supports the anti-arrhythmic role of the M_3_ receptor, its contributions to the action of the vagus nerve remain to be elucidated.

### Heart rate reduction and ventricular refractoriness

An obvious consideration when stimulating the vagus nerve is the resulting bradycardia. Some studies suggest that the anti-arrhythmic influence of the vagus nerve is reduced but not abolished when the heart rate is controlled [51, 52, 56, 58]. We have shown that the anti-VF effect of VNS is preserved during constant pacing in the isolated innervated rabbit heart [38]. In addition, we have shown that despite differential heart rate effects of left and right VNS, the anti-fibrillatory effect is equipotent [38]. The mechanisms by which a reduction in heart rate could protect from ventricular arrhythmias is not understood, but may include rate-dependent alterations in ventricular APD, refractoriness and dispersion of both factors. It is widely recognised that VNS prolongs ventricular APD and ERP [38, 104, 112, 131]. The direction of change in ERP during autonomic nerve stimulation mimics the change in VFT [38]. Using optical mapping of ventricular APD, we have shown that bilateral VNS reverses the sequence of ventricular repolarisation [113] although it is not clear how this might be cardioprotective. Detailed study on the effects of VNS on ventricular repolarisation is clearly needed. A similar anti-fibrillatory action has been noted when porcine hearts are treated with Ivabradine, an inhibitor of the pacemaker current (I_f_), [114] though it is not clear whether this is a consequence of induced bradycardia or from non-specific effects on other ion channels [115].

A common finding of clinical trials on HF is a negative correlation between heart rate and patient survival and the ability to increase heart rate in response to exercise [116]. The relationship between heart rate and mortality holds true in untreated patients and in those receiving β-blockers, although a reduction in heart rate and risk is seen in the latter. High heart rate is associated with cardiovascular risk by a number of independent factors (e.g. increased ventricular wall stress, decreased arrhythmic threshold) and factors which are related to adrenergic hyperactivity (i.e. left ventricular hypertrophy, lower arrhythmic threshold) [116]. VNS provides a mechanism, in addition to β-blockade, to reduce heart rate in these high risk patients; however, a large reduction in heart rate may not be ideal as excessive bradycardia carries its own potential risk. As with other studies, it is difficult to assess the relative contribution of different factors (i.e. heart rate reduction and direct electrophysiological effects) to the overall risk of higher heart rates, especially in regards to β-blockade and VNS with their multiple sites of action.

The relative importance of bradycardia as a protective mechanism is unclear and contrasts with our findings that the effects of VNS are preserved during constant ventricular pacing. VNS still has a profound effect on ventricular electrophysiology during constant pacing, reducing susceptibility to VF [38]. Studies conducted in 1952 by Silvo Weidmann [117] first demonstrated the role of time course of repolarisation in the excitability of Purkinje fibre and demonstrated that the membrane potential needed to return to at least -55 mV, following a triggered action potential, before another action potential could be elicited [117]. The electrophysiological implication of this is that factors (i.e. pharmacological, physiological) which shorten the time course of repolarisation are likely to be arrhythmogenic. Studies using anoxia and acetylcholine, the latter in atrial tissue, support this conclusion, accelerating repolarisation and promoting arrhythmia generation [118]. Increases in ERP, occurring without significant changes in conduction velocity, may provide protection from re-entrant arrhythmias although this may only be true if APD prolongation is uniform across the ventricular surface, as appears to be the case during VNS in the dog [112] but not in our studies in the innervated rabbit heart [113]. Several class III anti-arrhythmic compounds, such as amiodarone and sotalol, prolong phase 3 of the cardiac action potential and are recognised to reduce the occurrence of atrial and ventricular arrhythmias [119], although these compounds affect several different aspects of cardiac electrophysiology.

Another important concept in the generation of ventricular arrhythmias is that of action potential duration dispersion. Spatial repolarisation heterogeneity provides a substrate for the generation of ventricular arrhythmias, and dispersion of APD is a known mechanism underlying circus movement re-entry around refractory regions [120]. Although previous investigators have noted that the effects of VNS on electrophysiology are uniformly distributed [112], with no effects of APD dispersion, it is known that the SNS promotes a greater degree of APD shortening in basal regions of the ventricle [121], data which are supported by histological studies of sympathetic nerve distribution [122, 123]. When there is a background sympathetic tone, VNS may prevent arrhythmias by antagonising sympathetic mediated changes in spatial repolarisation (see accentuated antagonism). This idea may be particularly relevant in HF where there is an increased activation and influence of the sympathetic nervous system.

### Nitric oxide

The biosynthesis and multitude of biological functions of nitric oxide (NO) have been reviewed extensively elsewhere [123–127]. Our discussion will focus on the role of NO in relation to the parasympathetic nervous system.

#### Direct NO-dependent effects on arrhythmia susceptibility

It is widely accepted that NO is involved in both central and peripheral aspects of vagal control, not only in terms of cardiac function but also in other bodily systems such as the GI tract [128]. In the heart, NO appears to modulate the actions of the parasympathetic nervous system, regulating the change in HR in response to parasympathetic stimulation [129]. Herring et al. [130] suggested that NO acts pre-synaptically to reduce the bradycardic response to VNS. Other investigators have noted that NO modulates changes in A-V conduction in response to VNS in a similar manner [129] and suggests that NO has a role in the ventricular effects of VNS. We directly tested this possibility using the innervated rabbit heart.

**Fig. 3:**
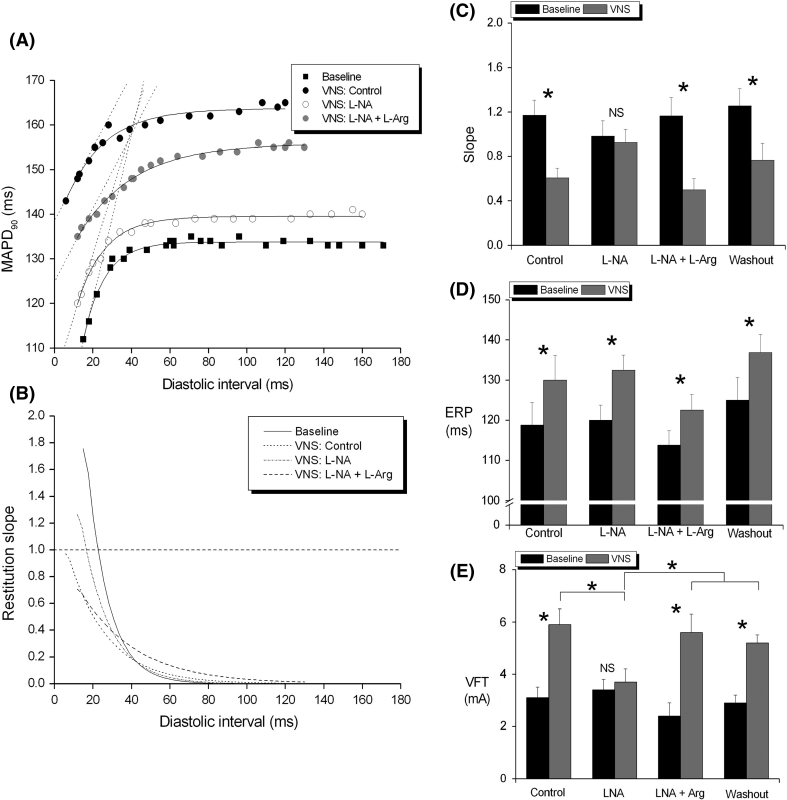
A direct nitric oxide synthase(NOS)–dependent anti-arrhythmic influence of vagus nerve stimulation. NOS-dependent anti-arrhythmic influence of vagus nerve stimulation (VNS) in the isolated innervated rabbit heart. **a** and **b** Data from a single experiment demonstrating how the influence of VNS on the restitution slope is abolished by perfusion with L-NA and restored by l-arginine (L-Arg). **c**, **d** and **e** Mean data effects of L-NA and L-NA + L-Arg on VNS induced changes in restitution slope, effective refractory period (ERP) and ventricular fibrillation threshold (VFT), respectively. *P < 0.05; comparison to baseline or between groups. Reproduced with permission from Brack et al. [104]

After confirming the anti-VF effect of VNS together with an increase in ERP and reduction in the slope of the restitution curve, we perfused the innervated heart preparation with the non-specific nitric oxide synthase (NOS) inhibitor L-NA and investigated the effects of VNS. In the presence of L-NA, the increase in VFT and flattening of electrical restitution slope with VNS were abolished whilst the increase in ERP was preserved [131] (see Fig. 3). The response to VNS was restored by supplementation with l-Arginine, the NO substrate which competes with L-NA for binding to NOS. This anti-arrhythmic effect of NO is supported by the work of Kumar et al. [132], who demonstrated that exogenous intrapericardially applied nitroglycerine significantly reduced the occurrence of ischaemia-induced VF. We later showed that NO donor, sodium nitroprusside, increased VFT, ERP and reduced the slope of the restitution curve in a similar manner to VNS [133]. These data tentatively show that (1) electrical restitution is important as a mechanism during VNS protection from VF as the effects of restitution and VFT parallel one another, (2) vagally mediated changes in perfusion pressure, heart rate and ERP do not appear to be significantly involved in vagal protection against VF as the changes are disproportionate in comparison with the changes in VFT and restitution, and (3) NO plays a key role in this protective effect.

**Fig. 4:**
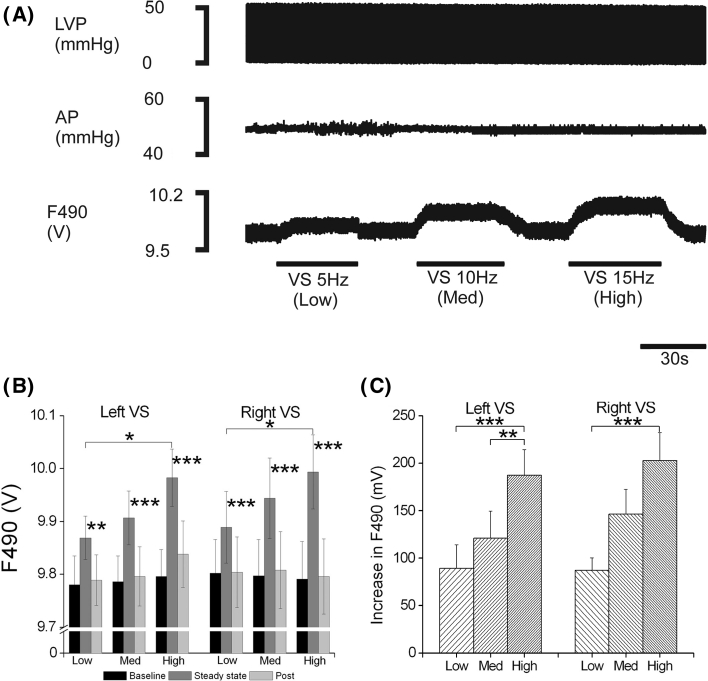
Direct measurement of NO release during vagus nerve stimulation. Measurement of nitric oxide release during cervical vagal nerve stimulation (VS), using the fluorescent indicator DAF2-DA, in the isolated innervated rabbit heart. **a** Raw data from a single experiment illustrating the change in *left ventricular pressure* (LVP), aortic perfusion pressure (AP) and nitric oxide dependent florescence (F490) during low-, medium- and high-intensity VS. **b** and **c** Mean data demonstrating the change in F490 before, during and after individual left and right VS. *P < 0.05, **P < 0.01, ***P < 0.001; comparison with steady-state response or between intensities of stimulation. Reproduced with permission from Brack et al. [134]

Although the aforementioned study provides basis for a working hypothesis for NO in mediating the ventricular effects of the vagus, it provided only indirect evidence of this mechanism. Through the use of the NO-sensitive dye DAF-2 DA, we were able to provide direct evidence of VNS-mediated NO release [134]. NO-dependent fluorescence was found to increase during both left and right cervical VNS in relation to the intensity of stimulation (see Fig. 4). Perfusion of L-NA significantly decreased background fluorescence, indicative of a reduction in basal NO production (presumably from the endothelium), and abolished the increase in fluorescence seen with VNS. TRIM, a specific antagonist of the neuronal form of NOS (nNOS or NOS I), had no effect on background fluorescence but abolished the fluorescence increase with VNS supporting the notion that NO production during VNS occurs through nNOS.

More recently, we have extended these findings to show that these effects of the vagus do not occur through activation of mAChR’s since perfusion with atropine at a concentration sufficient to block changes in heart rate and ERP did not influence the vagal effect on DAF-2 fluorescence, the reduction of the slope of the electrical restitution curve or increase in VFT [104]. In addition, this study indicates that the protective effects do not involve the endothelium (a substantial source of NO in the heart) as effects are preserved after functional endothelial denudation or during inhibition of vasoactive intestinal peptide (VIP) which is a neuroactive peptide released together with ACh.

It has previously been suggested that vagally released ACh and NO can act through parallel independent signalling pathways [135]. Given our evidence that NO production during VNS occurs through nNOS independent of the endothelium, VIP or ACh, we tentatively propose the existence of a separate network of parasympathetic–nitrergic anti-fibrillatory neurones [136] within the ventricle. It is known that nNOS is present in many parasympathetic neurones innervating the heart [137, 138], and there is also evidence of a subpopulation of intracardiac nerve fibres that contain solely NO coursing towards the ventricle in humans, providing supportive evidence for our hypothesis [139]. Given recent demonstrations of significant populations of parasympathetic innervation in the ventricles of mammalian species, refuting the classical view of sparse innervation, detailed neuro-anatomical information on the chemical phenotype of parasympathetic nerves within the ventricle is warranted.

#### VNS, NOS expression and NO signalling

NOS expression is found in endothelial cells, myocytes and neuronal cells, and it regulates a huge range of biological functions in a manner which appears to be dependent upon location [124–127]. The relative expression of the different NOS isoforms is altered significantly in both experimental and human HF, and dysfunction of NO signalling may be causally associated with many pathological changes associated with HF (i.e. reduced contractile function). Importantly studies conducted in dogs with microembolism-induced HF demonstrate that chronic VNS normalises both mRNA and gene expression of NOS [83]. Information on how alterations of NOS expression may alter ventricular arrhythmia susceptibility is currently lacking. Moreover, studies on the role on the NO-dependent anti-fibrillatory effects of VNS have not been carried out in failing hearts. Further studies are required to address the role of alterations in NO signalling and NOS expression in ventricular arrhythmia susceptibility and how VNS may modulate these mechanisms.

### Role of intracardiac neuronal networks and afferent nerve activation

It is recognised that direct neurochemical stimulation of decentralised intrinsic cardiac neurones can generate atrial and ventricular arrhythmias [42] and that the attenuation of parasympathetic control in HF may in part relate to changes in ganglionic function, as demonstrated in dogs with pacing induced HF [43]. Whether VNS can reverse these changes or modulate the intrinsic neuronal network to modify arrhythmia susceptibility is unknown.

Sensory (or afferent) nerve fibres relay information from the myocardium to the brain for processing, and approximately 70 and 80 % of the vagus nerve consists of these afferent fibres. Therefore, electrical stimulation of the vagus may capture both afferent and the effector efferent nerves, unless selective stimulation protocols, such as anodal block, are used to exclude a specific population of neurones. The contribution of these distinct populations of nerves to the anti-arrhythmic benefits of VNS is currently unknown. Studies conducted in our laboratory with the innervated rabbit heart show that the protective effect of VNS is not dependent upon the brain, but these experiments do not exclude the existence of additional anti-arrhythmic mechanisms in vivo or afferent reflex responses which can occur locally [38]. An earlier study rules out antidromic afferent nerve stimulation as a potential mechanism, since the response is lost during perfusion of the ganglionic transmission inhibitor hexamethonium. Myers et al. [52] demonstrated that the protective effects of VNS were maintained following decentralisation of the vagus nerve, but quantitative comparison of responses between groups was not carried out. Afferent nerve activation provides a, speculative, ‘explanation’ of the beneficial effects of chronic low-intensity VNS on haemodynamic parameters and remodelling in experimental HF (i.e. where no change of cardiac rate is seen) [84]. Afferent nerve activation and the modulation of central processes controlling autonomic outflow is a distinct possibility which requires attention.

### Role of vagal activated anti-inflammatory pathways

Another mechanism by which the parasympathetic nervous system may protect the heart is via an anti-inflammatory action. It has become increasingly evident over the two decades that inflammatory responses can be modulated and feedback into the autonomic nervous system, the so-called inflammatory reflex [140]. Locally released cytokines can activate sensory fibres which relay information to the central nervous system influencing efferent signals (refs). Vagotomy has a pronounced effect on the cytokine response to injury and ACh, acting through nicotinic ACh receptors, can inhibit the release of several cytokines [141]. The intracellular signalling pathways mediating this response have been reviewed by Li and Olshansky [142]. Interestingly, a strong inverse correlation between HRV and levels of inflammatory cytokines was report in a trial examining coronary artery risk in young adults [143]. Lower levels of cytokines are associated with a restricted inflammatory response in following MI and in HF, where it is recognised that arrhythmias contribute to a significant proportion of deaths.

The role of inflammation in the generation of arrhythmias in HF and following ischaemic injury is not clear, but it is recognised that malignant arrhythmias are more likely in patients with acute and chronic myocarditis [144, 145]. Cytokines can exert a direct action on myocyte electrophysiology but can also indirectly increase the susceptibility of the heart to arrhythmias by promoting the formation of non-conductive scar and fibrotic tissue, hypertrophy and atrophy of myocytes and impairment of oxygen availability [146]. Mice engineered to over-express tumour necrosis factor-α have been commonly used as a model of congestive HF, exhibiting a large number of ion channel conductance abnormalities and increased susceptibility to induced arrhythmias compared to wild-type controls [147–149]. Animals with TNF-α-induced failure exhibit a number of different types of arrhythmia, included atrial tachycardia, AF, non-sustained VT and ventricular ectopy, have altered repolarisation characteristics and dysregulated intracellular calcium homoeostasis [147]. Further study is warranted into the role of cytokines in arrhythmias and the potential role of the vagus in modulating this process. More detailed information on how inflammatory pathways may modulate ventricular electrophysiology is required and represents an area of some interest. It is possible that these studies could provide novel targets for the treatment for ventricular arrhythmias in HF and other arrhythmogenic conditions (i.e. long QT syndrome). It is recognised that inflammatory pathways may play an important role in the remodelling of the ventricles in HF [146] and that VNS can prevent/normalise ventricular phenotype in conjunction with the suppression of inflammatory activation. The structural abnormalities associated with ventricular remodelling (i.e. fibrosis and hypertrophy) may contribute to increased arrhythmic risk, and it is likely that VNS-mediated suppression of these pathways is a contributing factor to the anti-arrhythmic action of chronic VNS therapy in HF. This idea differs from the direct role of inflammatory mediators in arrhythmia susceptibility.

## Conclusions

The profound anti-arrhythmic influence of the vagus nerve has provided modern clinical practice with valuable tools for assessing risk in the form of HRV and BRS, and chronic VNS may prove to be a powerful therapy for heart failure. The effects of the vagus nerve on ventricular arrhythmogenesis are multi-faceted involving effects on heart rate, interactions with the sympathetic nervous system, anti-inflammatory influences and a direct effect on ventricular electrophysiology. Our data indicate that the vagus nerve exerts a direct anti-arrhythmic action at the level of the ventricles through the release of NO—a tentative-independent nitrergic neural network regulating cardiac physiology which could potentially offer a novel therapeutic avenue.
